# Supplementary material for: The Utility of Faecal Calprotectin, Lactoferrin and Other Faecal Biomarkers in Discriminating Endoscopic Activity in Crohn’s Disease: A Systematic Review and Meta-Analysis
Source: Biomedicines. 2023 May 9;11(5):1408. doi: 10.3390/biomedicines11051408 (PMC10216423; doi:10.3390/biomedicines11051408)
Supplement: Supplementary file 1 [file biomedicines-11-01408-s001.zip › biomedicines-2378196-supplementary.docx]

Supplementary

Table S1: FC to CD endoscopic activity dataset

| Author | Endoscopic activity definition | Endoscopically active CD patients | FC cut off | TP | FN | FP | TN |
| --- | --- | --- | --- | --- | --- | --- | --- |
| Bodelier 2017 | SES-CD =< 3 | 19 | 250 | 14 | 5 | 4 | 27 |
| Bjokesten 2021 | SES-CD =<2 | 103 | 94 | 87 | 16 | 6 | 17 |
| Buisson 2018 | presence of ulcers | 23 | 250 | 21 | 2 | 13 | 18 |
| Buisson 2021 | presence of ulcers | 44 | 250 | 37 | 7 | 10 | 29 |
| Chen 2017 | SES-CD =<2 | 35 | 250 | 34 | 1 | 6 | 15 |
| D'heans 2012 | presence of ulcers | 64 | 250 | 33 | 31 | 4 | 19 |
| D'Inca 2006 | SES-CD =< 3 | 23 | 80 | 19 | 4 | 1 | 4 |
| Dheans 2020 | SES-CD =<2 | 183 | 250 | 125 | 58 | 7 | 57 |
| Dheans 2020 | SES-CD =<2 | 48 | 50 | 36 | 12 | 7 | 26 |
| Falvey 2015 | SES-CD =<2 | 80 | 125 | 57 | 23 | 8 | 20 |
| Han 2022 | pSES-CD = 0 | 193 | 156.09 | 151 | 42 | 10 | 51 |
| Iwamoto 2018 | eSES-CD = 0 | 61 | 92 | 57 | 4 | 1 | 7 |
| Jesue 2015 | SES-CD = 0 | 28 | 54 | 20 | 8 | 6 | 18 |
| Karczewski 2015 | CDEIS <3 | 50 | 76 | 48 | 2 | 1 | 4 |
| Langhorst 2006 | Authors score | 33 | 48 | 27 | 6 | 2 | 8 |
| Lobaton 2013 | CDEIS <3 | 75 | 274 | 57 | 18 | 1 | 39 |
| Monisuszko 2017 | SES-CD =< 3 | 39 | 238.5 | 27 | 12 | 2 | 14 |
| Nancey 2013 | SES-CD =<2 | 38 | 250 | 27 | 11 | 9 | 31 |
| Penna 2020 | SES-CD =<2 | 53 | 155 | 51 | 2 | 15 | 12 |
| Reinisch 2020 | CDEIS =<3 | 76 | 250 | 51 | 25 | 8 | 71 |
| Schaffer 2014 | SES-CD =< 3 | 85 | 250 | 64 | 21 | 12 | 39 |
| Schoepher 2010 | SES-CD =< 3 | 114 | 70 | 101 | 13 | 7 | 19 |
| Sipponen 2008 | CDEIS =<3 | 52 | 200 | 49 | 3 | 21 | 33 |
| Swaminathan 2022 | SES-CD =<2 | 62 | 58.24 | 54 | 8 | 15 | 23 |
| Ye 2017 | SES-CD =<2 | 70 | 213 | 53 | 17 | 13 | 26 |
| Zollner 2021 | SES-CD =<2 | 31 | 78.4 | 28 | 3 | 3 | 10 |

Table S2: FC to CD mucosal healing activity dataset

| Cannatelli 2020 | SES-CD =<2 | 8 | 96 | 6 | 2 | 5 | 28 |
| --- | --- | --- | --- | --- | --- | --- | --- |
| Castiglione 2022 | SES-CD =<2 | 52 | 94 | 49 | 3 | 10 | 56 |
| Han 2022 | pSES-CD = 0 | 27 | 117.48 | 24 | 3 | 55 | 172 |
| Inokuchi 2016 | SES-CD=0 | 23 | 180 | 20 | 3 | 14 | 34 |
| Kawashima 2017 | SES-CD=0 | 16 | 162.2 | 13 | 3 | 11 | 51 |
| Reinisch 2020 | CDEIS =<3 | 80 | 250 | 72 | 8 | 25 | 51 |
| Vazquez-Moron 2017 | SES-CD =<2 | 22 | 71 | 21 | 1 | 23 | 26 |
| Noh 2020 | no ulcer or inflammation | 113 | 234 | 95 | 18 | 59 | 96 |
| Lopes 2018 | SES-CD=0 | 19 | 100 | 17 | 2 | 4 | 6 |

Table S3: FL to CD endoscopic dataset

| Author | Endoscopic activity definition | Endoscopically active CD patients | FL cut off | TP | FN | FP | TN |
| --- | --- | --- | --- | --- | --- | --- | --- |
| D'Inca 2006 | SES-CD =< 3 | 23 | 0.007 | 18 | 5 | 1 | 4 |
| Langhorst 2006 | Authors score | 33 | 7.25 | 27 | 6 | 4 | 6 |
| Sipponen 2008 | CDEIS =< 3 | 68 | 10 | 45 | 23 | 3 | 33 |
| karczewski 2015 | CDEIS <3 | 50 | 25 | 38 | 12 | 1 | 4 |

Table S4: Literature search strategy

| **Search Strategy** |
| --- |
| **Ovid Embase Search Strategy (As of 08 Aug 2022)**  1              inflammatory bowel disease/ or Inflammatory Bowel Diseases/  47591  2              (inflammatory adj2 bowel adj2 disease*).mp.     110624  3              Colitis, Ulcerative/ or ulcerative colitis/   84576  4              ((gravis or ulcerative or idiopathic) adj2 (colitis or proctocolitis)).mp.        94439  5              crohn disease/  100923  6              (crohn* adj2 (disease* or enteritis)).mp.               114242  7              enteriris/ or (granulomatous adj2 (colitis or enteritis)).mp.           727  8              (regional adj2 enteritis).mp.       393  9              (terminal adj2 ileitis).mp.             579  10           ileocolitis.mp.    661  11           1 or 2 or 3 or 4 or 5 or 6 or 7 or 8 or 9 or 10          213115  12           ((faecal or fecal) adj2 calprotectin).mp.  6474  13           fcal.mp.                354  14           feces/   68885  15           lactoferrin.mp. or lactoferrin test kit/ or lactoferrin/         13647  16           pyruvate kinase/ or M2-PK.mp. 10849  17           (M2 adj2 pyruvate adj2 kinase).mp.         1771  18           m2 pyruvate kinase/      28  19           biomarker.mp. or biological marker/       514844  20           14 and 19             2300  21           neopterin/ or neopterin.mp.      5990  22           myeloperoxidase/ or MPO.mp. or myeloperoxidase.mp. 48991  23           microRNA/ or MicroRNA.mp.      213208  24           alpha 1 antitrypsin/ or alpha-1 antitrypsin.mp.   21064  25           calcium binding protein/ or protein S 100/ or S100A12.mp.           40719  26           12 or 13 or 14 or 15 or 16 or 17 or 18 or 20 or 21 or 22 or 23 or 24 or 25 422774  27           diagnostic test accuracy study/  187479  28           diagnostic accuracy/       285222  29           Diagnosis.kw.    71565  30           false discovery rate/       2660  31           false negative result/      23029  32           false positive result/       36770  33           specificity.mp.   1009680  34           sensitivity.mp.  1670485  35           "sensitivity and specificity"/        439014  36           27 or 28 or 29 or 30 or 31 or 32 or 33 or 34 or 35               2349563  37           11 and 26 and 36               2277 |
| **Ovid Medline Search Strategy: (As of 08 Aug 2022)**  1 inflammatory bowel disease/ or Inflammatory Bowel Diseases/ 28391  2 (inflammatory adj2 bowel adj2 disease*).mp. 64228  3 Colitis, Ulcerative/ or ulcerative colitis/ 38926  4 ((gravis or ulcerative or idiopathic) adj2 (colitis or proctocolitis)).mp. 55576  5 crohn disease/ 42968  6 (crohn* adj2 (disease* or enteritis)).mp. 62179  7 enteriris/ or (granulomatous adj2 (colitis or enteritis)).mp. 627  8 (regional adj2 enteritis).mp. 1088  9 (terminal adj2 ileitis).mp. 498  10 ileocolitis.mp. 442  11 1 or 2 or 3 or 4 or 5 or 6 or 7 or 8 or 9 or 10 128861  12 ((faecal or fecal) adj2 calprotectin).mp. 2401  13 fcal.mp. 94  14 feces/ 100529  15 lactoferrin.mp. or lactoferrin test kit/ or lactoferrin/ 9461  16 pyruvate kinase/ or M2-PK.mp. 6974  17 (M2 adj2 pyruvate adj2 kinase).mp. 1035  18 m2 pyruvate kinase/ 0  19 biomarker.mp. or biological marker/ 462027  20 leukocyte L1 antigen complex/ 2961  21 14 and 19 2701  22 neopterin/ or neopterin.mp. 3766  23 Peroxidase/ or Myeloperoxidase.mp. or "MPO".ti,ab. 37256  24 microRNA/ or MicroRNA.mp. 135091  25 alpha 1 antitrypsin/ or alpha-1 antitrypsin.mp. 14478  26 calcium binding protein/ or S100A12 Protein/ or S100A12.mp. 28730  27 Occult Blood/ 6158  28 12 or 13 or 14 or 15 or 16 or 17 or 18 or 21 or 22 or 23 or 24 or 25 or 26 334907  29 sensitiv*.ti,ab. 1567966  30 diagnostic equipment/ 593  31 Diagnosis.kw. 40387  32 diagnos*.ti,ab. 2837660  33 diagnosis/ 17517  34 diagnostic errors/ 39408  35 specificity.mp. 1125100  36 (test adj2 accuracy).ti,ab. 5928  37 "sensitivity and specificity"/ 365550  38 29 or 30 or 31 or 32 or 33 or 34 or 35 or 36 or 37 4831257  39 11 and 28 and 38 1961 |
| **Pubmed Search Strategy: (As of 08 Aug 2022)**  ("Inflammatory Bowel Diseases/analysis"[Mesh] OR "Inflammatory Bowel Diseases/diagnosis"[Mesh] OR "inflammatory bowel disease"[Text Word] OR "crohn disease"[Mesh] OR "crohn's disease"[Text Word] OR "colitis, ulcerative"[Mesh] OR "ulcerative colitis"[Text Word] OR "colitis gravis"[Text Word] OR (idiopathic[All Fields] AND ("proctocolitis"[MeSH Terms] OR proctocolitis[Text Word] OR colitis[MeSH Terms] OR colitis[Text Word])) OR (crohn[All Fields] AND ("enteritis"[MeSH Terms] OR enteritis[Text Word])) OR (granulomatous[Text Word] AND (colitis[Text Word] OR enteritis[Text Word])) OR ileocolitis[Text Word] OR "terminal ileitis"[Text Word] OR "regional ileitis"[Text Word] OR "regional ileitis"[Text Word]) AND ((("feces/diagnosis"[Mesh] OR "feces/chemistry"[Mesh]) AND "biomarkers"[Mesh]) OR ((faecal[Text Word] OR fecal[Text Word]) AND (calprotectin[Text Word] OR "biomarkers"[Text Word])) OR "leukocyte L1 antigen complex"[Mesh] OR ("neopterin"[Mesh] OR "neopterin"[Text Word]) OR ("lactoferrin"[Mesh] OR "lactoferrin"[Text Word]) OR ("pyruvate kinase"[Mesh] OR "pyruvate kinase"[Text Word] OR "M2 pyruvate kinase"[Text Word]) OR ("alpha 1-Antitrypsin"[Mesh] OR "alpha 1-Antitrypsin"[Text Word]) OR ("Peroxidase"[Mesh] OR Myeloperoxidase[Text Word] OR "MPO"[All Fields]) OR ("S100A12 Protein"[Mesh] OR "S100A12"[All Fields] OR "calcium binding protein"[All Fields]) OR ("MicroRNAs"[Mesh] OR "MicroRNAs"[All Fields]) OR "Occult Blood"[Mesh] OR "Fecal Occult Blood Test"[All Fields]) AND ((sensitiv*[Title/Abstract] OR "sensitivity and specificity"[MeSH Terms] OR diagnose[Title/Abstract] OR diagnosed[Title/Abstract] OR diagnoses[Title/Abstract] OR diagnosing[Title/Abstract] OR diagnosis[Title/Abstract] OR diagnostic[Title/Abstract] OR diagnosis[MeSH:noexp]) OR (diagnostic equipment[MeSH:noexp] OR diagnostic errors[MeSH:noexp] OR diagnostic services[MeSH:noexp]) OR specificity[Title/Abstract] OR "false positive"[Title/Abstract] OR "false negative"[Title/Abstract] OR "test accuracy"[Title/Abstract]) |

Table S5: Tabular presentation of QUADAS-2 results

| **Study** | **RISK OF BIAS** | | | | **APPLICABILITY CONCERNS** | | |
| --- | --- | --- | --- | --- | --- | --- | --- |
|  | **PATIENT SELECTION** | **INDEX TEST** | **REFERENCE STANDARD** | **FLOW AND TIMING** | **PATIENT SELECTION** | **INDEX TEST** | **REFERENCE STANDARD** |
| Af Bjokesten 2021 | ☺ | ? | ☺ | ? | ☹ | ☺ | ☺ |
| Bodelier 2017 | ☺ | ☹ | ? | ☺ | ☺ | ? | ☺ |
| Buisson 2021 | ☹ | ☺ | ☹ | ☺ | ☹ | ☺ | ☹ |
| Buisson 2018 | ☺ | ☺ | ☹ | ☺ | ☺ | ☺ | ☹ |
| Buisson 2016 | ☺ | ? | ☺ | ☺ | ☺ | ☺ | ☺ |
| Cannatelli 2020 | ☺ | ? | ? | ☺ | ☺ | ☺ | ☺ |
| Castiglione 2022 | ☹ | ? | ? | ☺ | ☹ | ☺ | ☺ |
| Chen 2017 | ☹ | ☺ | ☺ | ☺ | ? | ☺ | ☺ |
| D'Heans 2012 | ☺ | ? | ☹ | ☺ | ☺ | ? | ☹ |
| D'Heans 2020 | ? | ☺ | ? | ☺ | ? | ☺ | ☺ |
| D'Inca 2006 | ☺ | ? | ☺ | ? | ☺ | ? | ☺ |
| Falvey 2015 | ☺ | ☹ | ☺ | ☺ | ☺ | ☺ | ☺ |
| Han 2022 | ☺ | ? | ☺ | ☺ | ☺ | ☺ | ☺ |
| Inokuchi 2016 | ☺ | ☹ | ☺ | ☺ | ☺ | ☺ | ☺ |
| Iwamoto 2018 | ☺ | ☹ | ☹ | ☺ | ☺ | ☺ | ☹ |
| Jesue 2015 | ☺ | ☺ | ☹ | ☺ | ☺ | ? | ☺ |
| Karczewski 2015 | ☺ | ? | ☺ | ? | ☺ | ? | ☺ |
| Kawashima 2017 | ☺ | ☺ | ☺ | ☺ | ☺ | ☺ | ☺ |
| Langhorst 2006 | ☹ | ☹ | ☹ | ☺ | ☹ | ? | ☹ |
| Lobaton 2013 | ☺ | ☺ | ☺ | ☺ | ☺ | ☹ | ☺ |
| Lopes 2018 | ☺ | ? | ? | ☺ | ☺ | ☺ | ☺ |
| Monisuszko 2017 | ☹ | ☺ | ? | ? | ☹ | ☺ | ☺ |
| Nancey 2013 | ☺ | ☺ | ☺ | ☺ | ☺ | ☺ | ☺ |
| Noh 2020 | ☹ | ? | ☺ | ☺ | ☹ | ☺ | ☹ |
| Penna 2020 | ☹ | ? | ☺ | ☺ | ? | ☺ | ☺ |
| Reinisch 2020 | ☹ | ? | ☺ | ? | ☹ | ? | ☹ |
| Schaffer 2014 | ☺ | ? | ☺ | ☺ | ☺ | ☺ | ☺ |
| Schoepher 2010 | ☺ | ☹ | ☺ | ☺ | ☺ | ☺ | ☺ |
| Sipponen 2008 | ☹ | ☹ | ☺ | ☺ | ☹ | ☺ | ☺ |
| Swaminathan 2022 | ☺ | ☹ | ☺ | ☺ | ☺ | ☺ | ☺ |
| Vazquez-Moron 2017 | ☺ | ☹ | ? | ? | ☺ | ☹ | ? |
| Ye 2017 | ☹ | ☹ | ? | ? | ☹ | ☺ | ☺ |
| Zollner 2021 | ☺ | ☹ | ? | ? | ☺ | ☺ | ☺ |

☺Low Risk ☹High Risk ? Unclear Risk

#

# PRISMA 2020 Main Checklist

| **Topic** | **No.** | **Item** | **Location where item is reported** |
| --- | --- | --- | --- |
| **TITLE** |  |  |  |
| **Title** | 1 | Identify the report as a systematic review. | 1 |
| **ABSTRACT** |  |  |  |
| **Abstract** | 2 | See the PRISMA 2020 for Abstracts checklist |  |
| **INTRODUCTION** |  |  |  |
| **Rationale** | 3 | Describe the rationale for the review in the context of existing knowledge. | 1-2 |
| **Objectives** | 4 | Provide an explicit statement of the objective(s) or question(s) the review addresses. | 1-2 |
| **METHODS** |  |  |  |
| **Eligibility criteria** | 5 | Specify the inclusion and exclusion criteria for the review and how studies were grouped for the syntheses. | 2-4 |
| **Information sources** | 6 | Specify all databases, registers, websites, organisations, reference lists and other sources searched or consulted to identify studies. Specify the date when each source was last searched or consulted. | 2 |
| **Search strategy** | 7 | Present the full search strategies for all databases, registers and websites, including any filters and limits used. | supplementary appendix |
| **Selection process** | 8 | Specify the methods used to decide whether a study met the inclusion criteria of the review, including how many reviewers screened each record and each report retrieved, whether they worked independently, and if applicable, details of automation tools used in the process. | 3-4 |
| **Data collection process** | 9 | Specify the methods used to collect data from reports, including how many reviewers collected data from each report, whether they worked independently, any processes for obtaining or confirming data from study investigators, and if applicable, details of automation tools used in the process. | 3 |
| **Data items** | 10a | List and define all outcomes for which data were sought. Specify whether all results that were compatible with each outcome domain in each study were sought (e.g. for all measures, time points, analyses), and if not, the methods used to decide which results to collect. | 3-4 |
|  | 10b | List and define all other variables for which data were sought (e.g. participant and intervention characteristics, funding sources). Describe any assumptions made about any missing or unclear information. | 3-4 |
| **Study risk of bias assessment** | 11 | Specify the methods used to assess risk of bias in the included studies, including details of the tool(s) used, how many reviewers assessed each study and whether they worked independently, and if applicable, details of automation tools used in the process. | 3 |
| **Effect measures** | 12 | Specify for each outcome the effect measure(s) (e.g. risk ratio, mean difference) used in the synthesis or presentation of results. | 3-4 |
| **Synthesis methods** | 13a | Describe the processes used to decide which studies were eligible for each synthesis (e.g. tabulating the study intervention characteristics and comparing against the planned groups for each synthesis (item 5)). | 3 |
|  | 13b | Describe any methods required to prepare the data for presentation or synthesis, such as handling of missing summary statistics, or data conversions. | 3-4 |
|  | 13c | Describe any methods used to tabulate or visually display results of individual studies and syntheses. | 6-13 |
|  | 13d | Describe any methods used to synthesize results and provide a rationale for the choice(s). If meta-analysis was performed, describe the model(s), method(s) to identify the presence and extent of statistical heterogeneity, and software package(s) used. | 3 |
|  | 13e | Describe any methods used to explore possible causes of heterogeneity among study results (e.g. subgroup analysis, meta-regression). | 3 |
|  | 13f | Describe any sensitivity analyses conducted to assess robustness of the synthesized results. | 3 |
| **Reporting bias assessment** | 14 | Describe any methods used to assess risk of bias due to missing results in a synthesis (arising from reporting biases). | 3 |
| **Certainty assessment** | 15 | Describe any methods used to assess certainty (or confidence) in the body of evidence for an outcome. | 3-4 |
| **RESULTS** |  |  |  |
| **Study selection** | 16a | Describe the results of the search and selection process, from the number of records identified in the search to the number of studies included in the review, ideally using a flow diagram. | 4-5 |
|  | 16b | Cite studies that might appear to meet the inclusion criteria, but which were excluded, and explain why they were excluded. | not applicable |
| **Study characteristics** | 17 | Cite each included study and present its characteristics. | 5-13 |
| **Risk of bias in studies** | 18 | Present assessments of risk of bias for each included study. | 5 |
| **Results of individual studies** | 19 | For all outcomes, present, for each study: (a) summary statistics for each group (where appropriate) and (b) an effect estimate and its precision (e.g. confidence/credible interval), ideally using structured tables or plots. | 5-13 |
| **Results of syntheses** | 20a | For each synthesis, briefly summarise the characteristics and risk of bias among contributing studies. | 5-13 |
|  | 20b | Present results of all statistical syntheses conducted. If meta-analysis was done, present for each the summary estimate and its precision (e.g. confidence/credible interval) and measures of statistical heterogeneity. If comparing groups, describe the direction of the effect. | 5-13 |
|  | 20c | Present results of all investigations of possible causes of heterogeneity among study results. | 5-13 |
|  | 20d | Present results of all sensitivity analyses conducted to assess the robustness of the synthesized results. | 5-13 |
| **Reporting biases** | 21 | Present assessments of risk of bias due to missing results (arising from reporting biases) for each synthesis assessed. | 5-13 |
| **Certainty of evidence** | 22 | Present assessments of certainty (or confidence) in the body of evidence for each outcome assessed. | 5-13 |
| **DISCUSSION** |  |  |  |
| **Discussion** | 23a | Provide a general interpretation of the results in the context of other evidence. | 11-14 |
|  | 23b | Discuss any limitations of the evidence included in the review. | 14 |
|  | 23c | Discuss any limitations of the review processes used. | 14 |
|  | 23d | Discuss implications of the results for practice, policy, and future research. | 14 |
| **OTHER INFORMATION** |  |  |  |
| **Registration and protocol** | 24a | Provide registration information for the review, including register name and registration number, or state that the review was not registered. | 2 |
|  | 24b | Indicate where the review protocol can be accessed, or state that a protocol was not prepared. | 2 |
|  | 24c | Describe and explain any amendments to information provided at registration or in the protocol. | 3-4 |
| **Support** | 25 | Describe sources of financial or non-financial support for the review, and the role of the funders or sponsors in the review. | not application |
| **Competing interests** | 26 | Declare any competing interests of review authors. | 15 |
| **Availability of data, code and other materials** | 27 | Report which of the following are publicly available and where they can be found: template data collection forms; data extracted from included studies; data used for all analyses; analytic code; any other materials used in the review. | supplementary appendix |

#####

# PRIMSA Abstract Checklist

| **Topic** | **No.** | **Item** | **Reported?** |
| --- | --- | --- | --- |
| **TITLE** |  |  |  |
| **Title** | 1 | Identify the report as a systematic review. | Yes |
| **BACKGROUND** |  |  |  |
| **Objectives** | 2 | Provide an explicit statement of the main objective(s) or question(s) the review addresses. | Yes |
| **METHODS** |  |  |  |
| **Eligibility criteria** | 3 | Specify the inclusion and exclusion criteria for the review. | Yes |
| **Information sources** | 4 | Specify the information sources (e.g. databases, registers) used to identify studies and the date when each was last searched. | Yes |
| **Risk of bias** | 5 | Specify the methods used to assess risk of bias in the included studies. | Yes |
| **Synthesis of results** | 6 | Specify the methods used to present and synthesize results. | Yes |
| **RESULTS** |  |  |  |
| **Included studies** | 7 | Give the total number of included studies and participants and summarise relevant characteristics of studies. | Yes |
| **Synthesis of results** | 8 | Present results for main outcomes, preferably indicating the number of included studies and participants for each. If meta-analysis was done, report the summary estimate and confidence/credible interval. If comparing groups, indicate the direction of the effect (i.e. which group is favoured). | Yes |
| **DISCUSSION** |  |  |  |
| **Limitations of evidence** | 9 | Provide a brief summary of the limitations of the evidence included in the review (e.g. study risk of bias, inconsistency and imprecision). | Yes |
| **Interpretation** | 10 | Provide a general interpretation of the results and important implications. | Yes |
| **OTHER** |  |  |  |
| **Funding** | 11 | Specify the primary source of funding for the review. | No |
| **Registration** | 12 | Provide the register name and registration number. | No |

*From:* Page MJ, McKenzie JE, Bossuyt PM, Boutron I, Hoffmann TC, Mulrow CD, et al. The PRISMA 2020 statement: an updated guideline for reporting systematic reviews. MetaArXiv. 2020, September 14. DOI: 10.31222/osf.io/v7gm2. For more information, visit: [www.prisma-statement.org](file:///Users/Anuj/Desktop/www.prisma-statement.org)
